# Ecological preferences and patient autonomy

**DOI:** 10.1136/jme-2024-110432

**Published:** 2024-12-05

**Authors:** Sabine Salloch

**Affiliations:** 1 Hannover Medical School, Hannover, Niedersachsen, Germany

**Keywords:** Decision Making, Environment, Ethics, Ethics- Medical, Informed Consent

## Abstract

Healthcare systems contribute considerably to worldwide carbon emissions and therefore reinforce the negative health impacts of climate change. Significant attempts to reduce emissions have been made on the macro level of politics and on the institutional level. Less attention has been paid so far to decisions that take place at the micro level of immediate doctor–patient contact. Current bioethical debates discuss potential tensions between ‘Green Healthcare’ and an orientation towards ethical principles such as promoting patient welfare or respect for patient autonomy. The article addresses this debate from a different angle starting from the premise that at least some patients might have a preference to reduce carbon outputs that are often deeply rooted in their personal value system. Taking different accounts of patient autonomy as a starting point, the article analyses whether such preferences must be respected as being part of patient autonomy. The analysis comes to a positive conclusion but highlights that certain factors such as misinterpretation, lack of understanding or pressure must be carefully considered. In addition, a patient’s climate-related preference does not per se justify the choice of treatment but must be integrated into shared decision-making and reconciled with the healthcare professional’s expert judgement on the intervention being a legitimate and promising way for reaching certain treatment goals. As a recommendation, empirical research on stakeholders’ attitudes, knowledge and practice regarding ecological sustainability in clinical decision-making is needed together with further ethical analyses.

## Climate change, human health and ethics

 Climate change’s implications for human health have gained growing attention in recent academic literature as well as in political and societal debates. Extensive research corroborates that climate change significantly increases health risks.^1 2^ The impacts are manifold ranging from rising temperatures that can lead to more heat-related illnesses to changing climate patterns that can contribute to the proliferation of disease vectors. As an additional consequence of climate change, air quality is deteriorating with exacerbation of respiratory and cardiovascular diseases.^3^ Further, climate-induced crises such as floods, wildfires and droughts have been linked to both direct injuries and long-term mental health effects.^4^ It becomes more and more obvious how the health of humanity is intricately tied to the health of the planet, requiring integrated policy responses to reduce greenhouse gas (GHG) emissions, enhance health resilience and mitigate the numerous health consequences of a changing climate.

Likewise, it is well known that healthcare systems themselves significantly contribute to global warming. The Lancet Countdown proposes that healthcare structures could be accountable for around 4.6% of total global GHG emissions.^5^ This is roughly equivalent to the emissions from the food sector, highlighting the magnitude of the healthcare sector’s carbon footprint.^6^ The emissions stem from various activities, particularly from energy use, waste disposal, pharmaceuticals and transportation. Research indicates that surgical and medical instrument manufacturing together with pharmaceutical preparation manufacturing alone can contribute up to 50% of the entire sector’s emissions.^7^ Importantly, the environmental impact varies significantly among different healthcare systems worldwide. High-income countries, such as the USA, possess the highest carbon footprints.

Significant attempts to reduce GHG emissions in healthcare have been made in some countries on the macro level of politics as well as on the institutional level with a special focus on the hospital sector. ‘Green Hospitals’—as a prominent catchphrase—aim to contribute to climate protection, for example, by critically assessing their consumption of electricity, water and construction materials as well as the use of medicinal products and devices.^8^ Less attention has been paid so far to decisions that take place at the micro-level of doctor–patient contact regarding the ecological impact of the treatments chosen.^9^ While it can be well argued that prospects for a significant reduction of GHG emissions are higher on the system and institutional levels, medical ethics should not neglect the role of ecological sustainability in the doctor–patient relationship. This is not least due to the fact that patients as well as doctors bring their ecological preferences with them to the clinical encounter where they (explicitly or implicitly) have a considerable influence on the decision-making process and its outcomes. There are also increasing attempts to find appropriate ways for introducing ecological aspects into shared decision-making, for example, through patient decision aids or various approaches to climate-sensitive health counselling.^9^

As the health impacts of ecological change become more and more visible, healthcare professionals have been called for action to serve as advocates for a ‘green’ transition in the healthcare sector.^10 11^ Such appeals are also documented in important guidelines of professional ethics such as the World Medical Association’s International Code of Medical Ethics and the Code of Ethics for Nurses of the International Council of Nurses. Christina Richie presents a four-principle-based framework for ‘Green Bioethics’^12^ and argues for the sharing of climate information with patients which offers them the options of lower-carbon choices (‘Green Informed Consent’).^13^ Resnik and Pugh, by contrast, hold that hat ‘there is not a generalisable duty to disclose environmental impact information to all patients during the consent process’ but that healthcare professionals should focus on advocating for system-level changes.^14^ In some sense, this article will address a complementary question within the debate on Green Informed Consent. It will start from the fact that (at least some) patients do have ecological preferences. Patients’ ecological preferences have only been sparsely addressed so far in the bioethical literature and elsewhere. The article analyses whether these preferences belong to patient autonomy and thus should be respected by healthcare providers. More concretely, the article discusses whether patients’ ecological preferences constitute reasons for healthcare professionals to initiate certain treatments that are wished for. Methodologically, the analysis draws on different accounts of patient autonomy and comes to the positive conclusion that ecological preferences can be legitimately considered as being an essential part of patient autonomy.

## Patients’ ecological preferences

At first glance, the question of whether patients’ ecological preferences are a component of patient autonomy might appear as rather non-controversial. Sure, one is tempted to say that these are preferences that need to be considered such as other preferences when it comes to informed consent or shared decision-making. At a closer look, however, it is far from being obvious which preferences need to be respected when patients wish for a certain therapy. In the negative case, legal standards usually guarantee that a treatment can be denied by a competent patient for any reason without the need to justify their decision (but with the right to be informed about the consequences of such a choice). Accordingly, a refusal is also valid for non-medical reasons such as in the case of Jehovah’s Witnesses and blood transfusions.

Positive non-medical preferences have been discussed less in the bioethics literature even if there is some research, for example, on patient preferences towards the person of the healthcare provider, for example, whether they are seen by a black or female doctor. How far such wishes need to be respected is a point of controversy.^15 16^ More examples and borderline cases, however, can be imagined. Think of the patient who wishes for outpatient treatment (instead of hospitalisation which is recommended) as she has to take care of her dog. Imagine the case of a patient demanding that his doctor has a certain age, gender or cultural background. Consider the case of a superstitious patient asking the doctor to wear red socks or a certain charm during the operation as she believes that this will contribute to the positive outcome of the intervention. Such claims are made by competent patients but are they part of patient autonomy that must be followed in its entirety? Or must they rather be seen as some other acts of self-expression from the patient’s side?

Usually, patient preferences are related to treatment and medical care. They contain ideas about which treatment is preferred (sometimes out of past experience) or they relate to statuses that the patient aims at or wants to avoid. However, there are also altruistic purposes that we regularly accept as a basis for medical interventions such as in blood or solid organ donations from living donors.^17^,^19^ There are also circumstances where we accept a patient taking a certain risk to serve public health benefits as is the case in vaccinations that do not primarily serve the individual but mainly prevent harm from other groups that might be affected by the disease more severely.^20 21^

If we now turn to patients’ ecological (or ‘climate-related’) preferences, the question comes up whether these preferences legitimise clinical decision-making to the same extent as—for example—preferences concerning the quality of life or the acceptance of risks in certain treatments. The question of whether to let ecological preferences have a say when patients express their treatment wishes gains topicality if we consider that a considerable share of the general population is worried about the deterioration of natural living conditions and strives to minimise their emissions in the private and professional contexts. According to the Eurobarometer survey more than three-quarters (77%) of European citizens think that climate change is a very serious problem (seriousness of climate change between 7 and 10 on a scale of 10).^22^ Regarding healthcare, a survey from the UK population found that 70% support the National Health Service goal to become the world’s first ‘net zero’ healthcare provider by 2045.^23 24^ At the same time, a survey of the German population found that most participants (65.1%) preferred to talk about their personal health in doctor–patient conversation as opposed to the health of all people who might be affected by ecological changes.^25^ Furthermore, a focus group study found that physicians might underestimate patients’ interest in discussing the environmental impacts of health decisions.^26^ Even if sound international evidence on patients’ climate-related preferences is widely missing, it must be expected that some (but not all) patients might have preferences regarding the carbon footprint of their medical treatments—a topic that they potentially introduce in medical consultations (or refrain from doing so as they find it inappropriate or anticipate adverse reactions from the doctor’s side). In addition, it needs to be considered that there are groups of patients for whom the protection of nature and climate is not a mere ‘preference’ but deeply rooted in their identity, for example, as indigenous people or environmental activists.

When discussing the significance of patients’ climate-related preferences, we could think of scenarios, for example, in which a decision is made about an optimal versus a less effective treatment that, however, appears as beneficial in light of carbon emissions. Examples that have already been discussed in this respect relate to inhalers for pulmonary disease or the use of anaesthetic gases.^27 28^ Trade-offs may also become necessary between cost-intensive and less costly options that differ in their ecological profile.^29^ Decisions could further concern treatments that are more risky but carbon-saving such as operating without robot assistance in cases where such assistance is already a validated and established procedure (eg, prostatectomy). Finally, there might be treatments that require more time and effort from the doctor’s (as well as from the patient’s) side but appear more climate-friendly. Here, we could think of a conservative versus an operative treatment of osteoarthritis, for example.

## Ecological preferences and patient autonomy

Recent publications have highlighted potential tensions between ‘Green Healthcare’ and an orientation towards powerful ethical principles such as promoting patient welfare or the respect for patient autonomy.^30 31^ In many cases, it currently seems to be ethically unclear whether healthcare providers are justified in offering treatments with a slightly limited quality, higher costs or an increased level of risk that are, however, preferable in their ecological footprints. If we consider this debate from a different angle—namely the premise that at least some patients might have the wish to reduce carbon outputs—this opens new perspectives for the normative evaluation which is then centred on the impact of such preferences for clinical decision-making. To better understand what such preferences might have to do with the norm to respect patient autonomy a brief look into different conceptions of autonomy as presented in the bioethical literature is helpful.

### Autonomy or liberty?

The debate on patient autonomy is a notoriously complex one with the term ‘autonomy’ being ‘loosely associated with several ideas, such as privacy, voluntariness, self-mastery, choosing freely, the freedom to choose, choosing one’s own moral position, and accepting responsibility for one’s choices.’^31^ (p. 7) One main trajectory of the debate lies in the distinction between a libertarian understanding of freedom of choice that patients possess as opposed to a more demanding, morally laden conception of autonomy that is often attributed to the Kantian tradition. Jennings comments in favour of the libertarian understanding by saying that ‘[i]ndeed, to talk about ‘autonomy’ in bioethics is something of a misnomer and a category mistake; the concept that is actually much more influential in bioethics is a version of Millian ‘liberty’, the version that Berlin dubbed ‘negative liberty’.^32^ (p. 73) There are more authors who doubt that a Kantian conception of autonomy as rational self-determination of the individual’s will is actually the moral framework that we mean when discussing respect for autonomy in clinical decision-making.^33^ (p. 30) ^34^ (p. 40)

Interestingly, patients’ preference to limit the carbon emissions of their treatments can be fruitfully interpreted from both rivalling theoretical perspectives. If we consider patient autonomy to be an issue of personal liberty that doctors must not interfere with searching for carbon-limited treatments is highly plausible as long as no interests of third parties are negatively affected. Usually, there will be no justifiable reasons to restrict patients’ liberty of placing emphasis on ecology-based considerations. If we hold, by contrast, that patient autonomy is related to a moral determination of the individual’s will (which is rather a minority position within bioethics) we can learn from the philosophical debate on climate ethics that there is a prima facie duty to care for the climate that must be expected from any human.^35^ If a patient adopts this position and—even in the case of personal suffering and risk—asks for a medical alternative that minimises carbon emissions for the benefit of public health and future generations, a morally demanding conception of autonomy provides good reasons for accepting this choice.

### Autonomy as authenticity or reflexivity

There are influential positions on patient autonomy that go beyond the liberty conception and intrinsically link the content of autonomous volitions to the person without necessarily defending a Kantian style autonomy. Gerald Dworkin in his earlier writings formulated the ‘authenticity’ condition of autonomy which refers to a person identifying with the influences that motivate them.^36^ Later on, Dworkin adds to this position by highlighting that it is not about the identification itself but about ‘the capacity to raise the question of whether I will identify with or reject the reasons for which I now act’.^37^ (p. 15) He also concedes that autonomy is a ‘global’ rather than a ‘local’ concept that evaluates a whole way of living rather than single decisions. Dworkin suggests that a necessary condition for being autonomous lies in the fact that a person’s second-order identifications are congruent with their first-order motivations.^36^ Together with the condition of independence, autonomy is mainly characterised by the fact that a person identifies with their wishes so that an authenticity of the self is warranted. Notoriously, this understanding of autonomy is related to theories of higher-order volitions such as Frankfurt’s compatibilist theory of free will.^38^ According to Frankfurt, ‘first-order desires’ to perform an action can become effective in a will. ‘Second-order desires’, that is, the desire to have a certain desire, can determine ‘second-order volitions’ which might change the concrete course of action. Therefore, a person exercises freedom of the will if they are free to want what they want to want.

More generally understood, interpreting autonomy as authenticity means that we take a reflective stance towards our own volitions. This affects our personality which critically relates to its impulses and either adopts them or not.^39^ Autonomy here has something to do with becoming reflective of which person we want to be. This is related to the volitions we adopt as being our own. Applied to patients’ wishes regarding the climate impact of their treatments, it can be easily argued that in most of the cases such preferences will be the product of a reflective process rather than emanating from mere affect or intuition. Even more, it is very likely that persons will have ‘first order volitions’ in many fields of their life (eg, food consumption or tourism) that are associated with a large carbon footprint. If they start relating critically to these impulses, they might however come to the conclusion that they do not really want what they initially wanted but—through reflection—their volitions might change for options that are more climate-friendly. If patients introduce such volitions in doctor–patient conversation, according to the authenticity view of autonomy, there are good reasons to respect them as the product of a reflective process in which the patient has critically examined their initial desires and has finally decided on certain options.

### Principlism

A standard view of patient autonomy, closely related to the practice of informed consent, builds on three ‘nonideal conditions’ as moral requirements.^40 41^ According to this ‘principlist’ view, autonomous actions occur intentionally with understanding and without controlling influences that would unduly determine them. Whereas actions are either intentional or non-intentional the other two criteria come in degrees: A patient’s understanding can be more or less developed and there may be from no to very powerful controlling influences on decision-making. Beauchamp and Childress thus declare that ‘it needs only a substantial degree of understanding and freedom from constraint, not a full understanding or a complete absence or influence’.^41^ (p. 101) The principlist account of autonomy focuses on actions rather than on the autonomous person. The authors aim to provide a non-ideal theory suitable for ‘normal choosers’ who act. They also discuss an abundance of borderline cases and situations that might be encountered in clinical practice, for example, when dealing with incompetent or marginally competent patients. In the principlist account, it is very well possible that competent choosers make non-autonomous judgments, for example, when they are heavily influenced by external factors or when they are not informed or ill-informed. The account gains its main attraction from its applicability to many different clinical settings. Even if the notions included (eg, intentionality, understanding, control) need to be further clarified, the absence of highly contested concepts such as freedom of the will or the concept of a person makes this account well applicable in practice.

Applied to the question of respect for patients’ ecological preferences, it emerges that each action needs to be evaluated individually to see how it aligns with the three conditions for autonomy. With the requirement of intentionality, there will usually not be a problem as it is very unlikely that ecological interests come up involuntarily or without any reflection. The other two criteria, however, are worth considering: Even if climate-sensitive health counselling emerges as a more and more important topic,^9^ ecological aspects do not play a role in the majority of healthcare consultations so far. It is not only that healthcare professionals are per se reluctant to inform about the ecological impact of treatments but sound evidence on such impact is very often missing. This might infringe on the criterion of ‘understanding’ but is not completely novel to healthcare and counselling. Dealing with limited evidence and ignorance forms a key aspect in many medical consultations and procedures and this experience could be transferred to counselling about the climate impact of certain treatments. It is worth considering, however, that patients might also receive misguiding information or information that they are not able to fully interpret by themselves. This is another reason to let climate effects become the subject of doctor–patient consultations in settings where this is appropriate.^13^ The requirement of no controlling influences, finally, in rare cases might also play a role with respect to climate-related inquiries. It must be precluded that the patient’s surroundings (eg, family or activist groups they are involved in) have an undue influence on their decision-making. There could be very subtle social sanctions that play a role for patients who need to undergo ‘high carbon’ treatments if they live in a particularly eco-sensitive environment. In summary, the principlist account of autonomy sheds an interesting light on respect for patients’ ecological preferences. Even if certain conditions (understanding, lack of control) need to be carefully considered, there is no general reason not to respect patients’ ecological preferences as being part of their autonomy.

### Summary and further perspectives

In sum, none of the accounts of patient autonomy discussed above precludes ecological preferences from being considered as part of patient autonomy in the same way as other preferences that might relate to quality or length of life or the acceptance of risks or burdens. Unlike other preferences that patients may have, however, ecological preferences predominantly do not relate to effects that the patient himself or herself will experience. Instead, they are rather motivated by a patient’s interest in public health which transcends immediate care in the spatial (as effects of climate change often take part in other regions of the world) and temporal (as future generations are affected) dimensions.

Importantly, however, patients’ ecological preferences cannot settle treatment decisions: Discussing a certain treatment option is always linked to a healthcare professional’s expert judgement about whether the intervention is a legitimate and promising way to reach certain treatment goals that are wished by the patient.^42^ Patients cannot demand a treatment for ecological reasons alone but a medical rationale is always needed. In clinical practice, however, there might be many instances in which different treatments (or the option of non-treatment) can at least be reasonably discussed against each other in light of many considerations—the ecological footprint being one of them.

To strengthen the understanding of the role that ecological considerations might play in clinical consultations, empirical research is needed on stakeholders’ experiences knowledge and perspectives. So far, we know very little about the frequency with which climate change plays a role in doctor–patient conversations and we also do not know how doctors use it to deal with respective requests. From a normative (medical ethics) perspective, it would be worth analysing, for example, which role a doctor’s ecological attitudes can legitimately play or whether even a medical paternalism in favour of ecological sustainability could be justified. The intersection between the (professional) normative commitment of doctors towards climate protection^10^ and patients’ interests that point in the same (or the opposite) direction should give rise to comprehensive ethical analyses clarifying the respective commitments and obligations.

In clinical practice, models for integrating climate aspects in shared decision-making should be developed for settings where this is appropriate. This might also include the education of patients who—due to ‘carbon innumeracy’—overestimate or underestimate certain effects. Admittedly, depending on the concrete healthcare system, place of residence, insurance status, health literacy and other factors the prospects for successfully implementing climate-sensitive health decisions will be more or less realistic. In any case, healthcare professionals should be sensitive about sustainability issues in cases where they are raised by the patients. They should also critically consider where to introduce the topic in an appropriate manner by themselves. Nothing speaks against healthcare professionals being advocates for ‘Planetary Health’^11^ but at the same time, they must uphold their duties towards the individual patient, their well-being and their autonomy.

## Data Availability

No data are available.
